# RApid Primary care Initiation of Drug treatment for Transient Ischaemic Attack (RAPID−TIA): study protocol for a pilot randomised controlled trial

**DOI:** 10.1186/1745-6215-14-194

**Published:** 2013-07-02

**Authors:** Duncan Edwards, Kate Fletcher, Rachel Deller, Richard McManus, Daniel Lasserson, Matthew Giles, Don Sims, John Norrie, Graham McGuire, Simon Cohn, Fiona Whittle, Vikki Hobbs, Christopher Weir, Jonathan Mant

**Affiliations:** 1 General Practice and Primary Care Research Unit, Institute of Public Health, University of Cambridge, Forvie Site, Robinson Way, Cambridge CB2 0SR, UK; 2 Department of Primary Care and General Practice, Clinical Sciences Building, The University of Birmingham, Edgbaston B15 2TT, UK; 3 Department of Primary Health Care, University of Oxford, Rosemary Rue Building, Old Road Campus, Headington, Oxford OX3 7LF, UK; 4 Stroke Prevention Research Unit, Department of Clinical Neurology, University of Oxford, Level 6, West Wing, John Radcliffe Hospital, Oxford OX3 9DU, UK; 5 Department of Geriatric Medicine, University Hospital Birmingham NHS Trust, Birmingham B29 6JD, UK; 6 Robertson Centre for Biostatistics, University of Glasgow, Glasgow G12 8QQ, UK; 7 Stroke Research Network, NIHR Stroke Research Network Coordinating Centre, Bioscience Centre, International Centre for Life, Times Square, Newcastle upon Tyne NE1 4EP, UK

## Abstract

**Background:**

People who have a transient ischaemic attack (TIA) or minor stroke are at high risk of a recurrent stroke, particularly in the first week after the event. Early initiation of secondary prevention drugs is associated with an 80% reduction in risk of stroke recurrence. This raises the question as to whether these drugs should be given before being seen by a specialist – that is, in primary care or in the emergency department. The aims of the RAPID-TIA pilot trial are to determine the feasibility of a randomised controlled trial, to analyse cost effectiveness and to ask: Should general practitioners and emergency doctors (primary care physicians) initiate secondary preventative measures in addition to aspirin in people they see with suspected TIA or minor stroke at the time of referral to a specialist?

**Methods/Design:**

This is a pilot randomised controlled trial with a sub-study of accuracy of primary care physician diagnosis of TIA. In the pilot trial, we aim to recruit 100 patients from 30 general practices (including out-of-hours general practice centres) and 1 emergency department whom the primary care physician diagnoses with TIA or minor stroke and randomly assign them to usual care (that is, initiation of aspirin and referral to a TIA clinic) or usual care plus additional early initiation of secondary prevention drugs (a blood-pressure lowering protocol, simvastatin 40 mg and dipyridamole 200 mg m/r bd). The primary outcome of the main study will be the number of strokes at 90 days. The diagnostic accuracy sub-study will include these 100 patients and an additional 70 patients in whom the primary care physician thinks the diagnosis of TIA is possible, rather than probable. For the pilot trial, we will report recruitment rate, follow-up rate, a preliminary estimate of the primary event rate and occurrence of any adverse events. For the diagnostic study, we will calculate sensitivity and specificity of primary care physician diagnosis using the final TIA clinic diagnosis as the reference standard.

**Discussion:**

This pilot study will be used to estimate key parameters that are needed to design the main study and to estimate the accuracy of primary care diagnosis of TIA. The planned follow-on trial will have important implications for the initial management of people with suspected TIA.

**Trial registration:**

ISRCTN62019087

## Background

The high risk of stroke following a transient ischaemic attack (TIA) or minor stroke is now well recognised, with an untreated risk of recurrence by 90 days around 18% [1,2]. Much of this excess risk is in the first few days following the initial event [3]. Simple clinical features (an ABCD2 score, based on age, blood pressure (BP), clinical features, diabetes and duration) can identify those people who are at particularly high risk in the first 7 days [4,5]. On this basis, NICE recommends that people at the highest risk of stroke are seen by a specialist within 24 hours of symptom onset (to maximise the chance that risk-modifying treatment is initiated before a further event) and people at lower risk of stroke are seen within 7 days. The National Institute for Health and Care Excellence (NICE) recommends that 300 mg aspirin daily is prescribed while awaiting specialist treatment, but no other secondary prevention treatment is recommended prior to confirmation of diagnosis [6].

Approximately 10,000 recurrent strokes occur within the first 90 days after TIA and minor stroke each year [7]. If rapid primary care initiation of secondary prevention drug treatment could reduce 90-day recurrence rates further from 10% to 2%, this would be equivalent to preventing 8,000 strokes per year. A reduction in the national stroke incidence in this 5% to 10% range would also lead to savings in care costs of the order of £90Mto £180Mper year in England alone, based upon 2005 National Audit Office calculations.

### Evidence that very early treatment reduces recurrent stroke

There is an emerging evidence base as to what treatments will reduce the risk of stroke in the immediate days and weeks following TIA. Aspirin is effective when given as soon as possible [8], and for people with significant carotid artery stenosis, carotid endarterectomy is highly effective as a secondary care surgical procedure: the number of patients that must be treated to prevent one ipsilateral stroke within 5 years’ follow-up is five within a fortnight of the initial stroke or TIA (compared to 125 after 12 weeks) [9].

There is evidence that cholesterol lowering and blood-pressure lowering are effective in the long term with regards to secondary stroke prevention [10,11], but there is uncertainty with regard to their value in the early phase. With regard to cholesterol lowering, there was no evidence of benefit of early administration of simvastatin in the pilot FASTER trial, which comprised patients with a TIA or minor stroke within 24 hours of onset who had been admitted to hospital [12]. Trials of blood-pressure lowering in the acute phase of stroke are ongoing, but their findings may be difficult to apply to people whose symptoms have resolved at the time of treatment initiation (that is, people who have had a TIA or minor stroke).

There is some evidence for dual anti-platelet therapy, with the pilot FASTER trial finding a lower risk of stroke in patients receiving clopidogrel plus aspirin compared to aspirin alone [12], though there was no evidence from the MATCH trial that in the longer term this combination was beneficial, with a significantly higher risk of life-threatening bleeds in the group on combination therapy [13]. There is, however, some evidence of longer-term dual anti-platelet therapy benefit from the ESPRIT and ESPS-2 trials, which found the combination of aspirin and dipyridamole was superior to aspirin alone for the treatment of people randomised within 6months of a TIA or minor stroke [14,15]. The EARLY study found a lower risk of stroke and a lower risk of a composite of stroke, TIA, myocardial infarction, major bleeding and death (hazard ratio 0.73, confidence interval 0.44 to 1.19) in patients started on aspirin and dipyridamole within 24 hours of initial symptoms versus patients started on aspirin within 24 hours with dipyridamole started after 7 days, although the results were not statistically significant [16].

Set against this randomised controlled trial (RCT) evidence of mixed benefit for early initiation of secondary prevention medications, there has been encouraging observational data of the beneficial effect of early use of these drugs from the EXPRESS and SOS-TIA studies [3,17]. In the EXPRESS study, changing the protocol ata specialist TIA clinic whereby secondary prevention drugs (dual anti-platelet therapy, blood-pressure lowering therapy and simvastatin) were commenced on the same day rather than waiting for the general practitioner (GP) to commence treatment on receipt of a clinic letter was associated with an 80% reduction in the risk of stroke at 90 days. The median delay from prescription of first treatment fell from 20 days to 1 day. In the SOS-TIA study, patients with suspected TIA (53% within 24 hours of symptom onset) were seen in a specialist clinic where a stroke prevention programme was initiated. The 90-day risk of stroke in patients treated in this way was about 80% lower than would have been anticipated given their clinical features by applying the ABCD2 score [5].

### Why is a trial of early treatment in primary care necessary?

Many patients with TIA suffer a stroke before they are seen by a specialist, and it is likely that some of these strokes are preventable by earlier initiation of secondary prevention by the first doctor to see the patient, either in primary care or an emergency department.

In the NORTHSTAR study set in five TIA clinics (with a median delay of 15 days from symptom onset to specialist review), the observed 90-day risk of stroke was less than half what it is in studies where patients were seen within the first day [18]. This supports the concept that patients are at highest risk in the period before they encounter a specialist, and therefore the most effective way of preventing a recurrent stroke is for the first doctor who sees the patient to give medication prior to specialist review.

Despite recent advances in access to TIA clinics, the national stroke audit, 2010, reports in its key messages that it is still difficult to achieve appropriate timely access for high risk patients in secondary care:

*High-risk patients are still not being seen quickly enough. A third of centres admit high-risk TIA in order to access specialist assessment, although we know that TIA patients generally sit outside of the acute stroke unit when admitted. Only 10% of centres provide a seven day a week outpatient based neurovascular clinic for high-risk TIA. Access to carotid imaging for high-risk TIA patients on the same day including the weekend is only possible in 10% of centres. As imaging is an integral part of specialist assessment its low level of provision undermines the benefit of admitting high-risk TIA patients. Almost half of centres admit low-risk TIAs, which is probably a wasteful use of resources*[19].

The proposed trial will investigate the role primary care physicians (PCPs) might play in ensuring rapid treatment – should they give immediate therapy, or simply ensure prompt referral? The pilot work for the trial will also help develop material of immediate NHS relevance – guidance to primary care physicians on how to diagnose TIA and data on the accuracy of primary care physician diagnosis.

Current NICE guidelines recommend that patients with suspected TIA or minor stroke should not routinely be admitted to hospital and should be assessed by a specialist within 7 days (or 24 hours for people at particularly high risk of stroke on the basis of their ABCD2 score) [6]. This trial will compare rapid primary care initiation of secondary prevention with the current UK standard care as described in the NICE guideline.

### Aims

The aims of the trial are to determine the feasibility of a randomised controlled trial, to analyse cost effectiveness analysis and to ask: Should primary care physicians initiate secondary preventative measures in addition to aspirin in people they see with suspected TIA or minor stroke at the time of referral to a specialist?

Questions to address:

1. Is randomisation at the individual patient level possible?

a. Is individual randomisation practical in the primary care environment?

a. Is there evidence of contamination? That is, what proportion and to what extent do patients randomised to usual care receive the active additional treatment?

2. What sample size (and what number of practices) is required for a future trial?

a. What is the recruitment rate of patients to the trial?

a. What is the follow-up rate throughout the trial?

a. What is the preliminary estimate of the primary event rate (number of strokes at 90 days)?

3. Are proposed outcome measures for a future trial feasible?

4. Werethere any adverse events?

5. How accurate is primary care physician diagnosis of TIA?

## Methods/Design

### Outline of design

**Figure 1:**
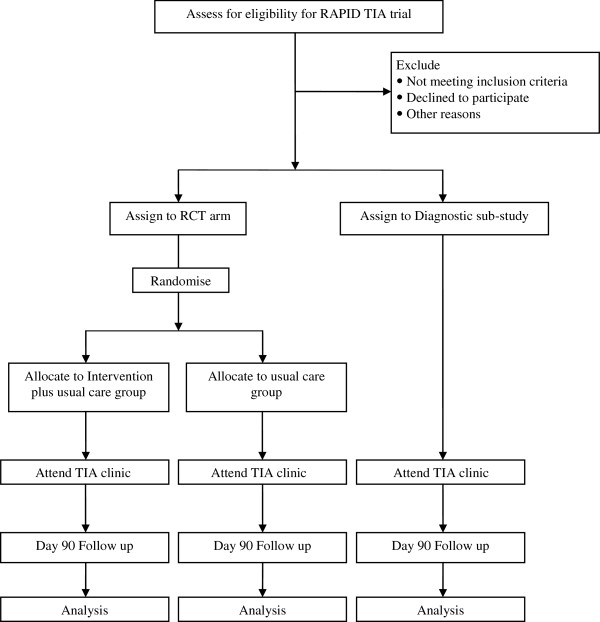
Cohort diagram of the RAPID-TIA trial.

A pilot trial will recruit patients with symptoms suggestive of TIA or minor stroke in general practices from the catchment of three hospital TIA clinics (Birmingham, Cambridge and Oxford) and the emergency department in Cambridge (Figure 1). Embedded within this pilot trial will be a diagnostic accuracy study comparing the initial diagnosis made by the primary care physician with the final diagnosis following review and investigation at the specialist clinic. When a primary care physician sees a patient with a history suggestive of TIA, are cord will be made of whether TIA or a minor stroke is probable or possible. All patients will then be referred to the TIA clinic. Probable cases will be invited by the primary care physician to enter the trial and the diagnostic accuracy study; possible cases will be invited to enter the diagnostic accuracy study only. Patients will be allocated by telephone to additional pre-TIA clinic drug treatment and usual care or usual care only, with allocation by a minimisation scheme using the following categories: TIA clinic catchment area, primary care clinic type and ABCD2 score. The TIA clinic will record the final diagnosis and adjust treatment accordingly. ‘Usual care’ implies initiation of aspirin therapy and referral to the specialist TIA clinic. The primary outcome will be the number of strokes at 90 days. Semi-structured interviews will be undertaken with the aim of determining how trial procedures might be modified for the main trial.

### Study population

The study population will comprise patients attending general practice, out-of-hours general practice and emergency departments with recent symptoms suggestive of TIA or minor stroke. The primary care physician will assess whether a diagnosis of TIA or minor stroke is probable or possible. The former group will be eligible for the trial. The latter group will not be eligible for the trial, but will be eligible for the diagnostic accuracy study.

### Identification of patients

Eligible patients will be drawn from general practices in the catchment areas of the TIA clinics in Queen Elizabeth Hospital (Birmingham), Walsall Manor Hospital (Birmingham), Addenbrooke’s Hospital (Cambridge) and John Radcliffe Hospital (Oxford), as well as the emergency department in Cambridge. Oxfordshire practices that are participating in the OXVASC study will be excluded from the trial. For the pilot trial, we aim to recruit approximately ten practices from each area and one out-of-hours general practice base from each area.

### Inclusion and exclusion criteria

#### Inclusion criteria

Patients presenting to a primary care physician with symptoms suggestive of TIA or minor stroke (first and recurrent, but only one event per patient in the trial).

#### Exclusion criteria

Patients will be excluded if:

• The PCP considers that immediate emergency admission is required for any reason. This will include patients where symptoms have not substantially resolved by the time they see the PCP. PCPs will record reasons for exclusion.

• The patient has experienced a TIA or stroke within the previous month.

• The PCP is not in equipoise.

### Recruitment

About 3 of the 1,000 people per annum who present with symptoms suggestive of TIA or stroke are not managed as inpatients [7]. Therefore, a practice with a list size of 7,500 will have 20 patients per year that might be referred into the study. If we assume that 5 people per practice per year are actually randomised, this would mean that 30 practices recruiting patients over a 1year period would recruit 150 patients. Additional patients will be recruited via out-of-hours general practice services and emergency departments to reach a recruitment target of 170, of whom we estimate 100 will be recruited to the trial, and an additional 70 patients to the diagnostic accuracy study only.

### Patient information and consent

Patients who are suspected of having a probable TIA or minor stroke will receive information sheet A and be invited to enter the pilot trial and diagnostic study. Patients who are suspected as having a possible TIA or minor stroke will receive information sheet B and be invited to enter the diagnostic study only. Patients will be given time to consider the information and to ask questions. If the patient declines to enter the pilot or diagnostic study, usual care will proceed. If the patient accepts the invitation, usual care will proceed alongside an assessment of eligibility for the trial. The primary care physician will obtain written consent from eligible patients who are willing to take part in either study.

### Randomisation

Telephone and internet randomisation will be available utilising the resources of the Robertson Centre for Biostatistics, part of the Glasgow Clinical Trials Unit. During the pilot phase, a minimisation scheme will be utilised across the whole trial, using the following minimisation factors: TIA clinic catchment area (Birmingham, Cambridge or Oxford), clinic type (general practice, out-of-hours general practice or emergency department) and risk of stroke using the ABCD2 score (4 or more; less than 4). When an eligible patient has given written consent to take part, the primary care physician will ring the dedicated randomisation number of the Clinical Trials Unit. Patient and site information will be given to the trials unit who will assign the patient to additional treatment plus usual care or usual care only.

### Sample size

This pilot study will be used to estimate the sample size required for a future RCT. The diagnostic accuracy study embedded within this pilot study requires a sample size of approximately 100 probable cases and 67 possible cases. GP diagnosis will be compared to TIA clinic diagnosis in the light of relevant investigations. We will not only be able to report on the predictive value of GP diagnosis, but also sensitivity and specificity since the ‘possible’ TIAs will also be referred to the clinic (although they will not take part in the clinical trial). If we have 100 probable and 67 possible cases referred, and assume sensitivity around 80%, predictive value of around 67% and specificity around 60% from our previous work on modelling the impact of different patterns of TIA services [20], then the confidence intervals will be between +/−10% for each of these parameters.

### Trial interventions

#### Intervention plus usual care group

Intervention group patients will be treated with additional secondary prevention medications, unless there are clinical contraindications, prior to referral to a specialist clinic. Treatment will comprise of dual anti-platelets, blood-pressure lowering medication, and simvastatin 40 mg. All patients will then be referred to a specialist clinic as per NICE guidelines (with the degree of urgency dictated by the ABCD2 score) [6].

#### Dual anti-platelets

In patients not already on anti-platelet or anti-coagulant therapy, aspirin 300 mg once daily will be started for the first 2 weeks or until reduced by a specialist. Dipyridamole MR 200 mg twice daily will be started in patients not already on anti-platelet or anti-coagulation therapy other than aspirin. Any anti-coagulation or anti-platelet agents the patient is already taking will be continued.

#### Blood-pressure lowering medication

Unless BP is below 130 mmHg systolic on either of two readings taken one minute apart or the patient is already taking all three of a thiazide diuretic, ACE-inhibitor and calcium channel blocker, then one of these three classes of medication will be initiated according to the PCP’s clinical choice. If the patient is already taking all three drug classes, one agent should have its dose increased within its licensed dosage according to the PCP’s clinical choice.

#### Simvastatin 40 mg once daily

Simvastatin 40 mg daily will be started unless the patient is already on statin treatment of equivalent intensity.

Clinicians should check liver function or renal function when starting statins or blood-pressure lowering medication according to their usual practice, but results are not mandatory prior to initiating treatment.

#### Usual care group

This is the control group. If patients are not already on anti-platelet therapy, a loading dose of aspirin will be administered (300 mg daily) and patients will be referred to a specialist clinic as per NICE guidelines (with the degree of urgency dictated by the ABCD2 score) [6]. All patients will be instructed to continue with their usual medication unless a clinician makes a specific decision to alter it for any reason.

### Diagnostic accuracy study

All patients who consent will be entered into the diagnostic study; patients with a diagnosis of possible TIA or minor stroke will be entered only into the diagnostic study.

The primary care physician will complete the usual local TIA clinic referral form. In addition, the PCP will record the presence or absence of symptoms, signs and past medical history relevant to the diagnosis of TIA, including individual features to enable the ABCD2 score to be calculated: age, blood pressure, clinical features including presence of unilateral weakness and speech impairment, duration of symptoms and diabetes [5]. The PCP will provide a level of certainty of diagnosis based upon clinical judgement: probable or possible.

All patients will then be referred to a specialist clinic as per NICE guidelines (with the degree of urgency dictated by the ABCD2 score) [6]. At the TIA clinic, the diagnosis, ABCD2 score and clinical features will be recorded. Patients will be asked to give consent that their clinical notes can be reviewed to ascertain their final diagnosis (as a final diagnosis may not be reached at the initial specialist appointment).

### Summary of procedures and staffroles

Throughout the trial, the primary care physicians will be instructed to follow usual practice, which should be consistent with the 2008 NICE stroke guidelines [6]. All patients with suspected TIA or minor stroke should be continued on their anti-platelet treatment or started on aspirin 300 mg daily unless there are contraindications and referred to a specialist clinic (with the degree of urgency dictated by the ABCD2 score) [6]. The intervention group will receive treatment additional to this usual care.

#### Initial presentation in daytime general practice

Participating general practices have a lead GP, who coordinates recruitment into the trial. Patients may present with suspected TIA at any time to a GP in the practice, who may be assisted in aspects of patient care by a practice nurse. When a patient presents, the clinician will take a history, examine the patient and come to an initial diagnosis of possible or probable TIA or minor stroke as per usual practice, and then:

1. Inform the patient of the suspected diagnosis as per usual practice.

2. Discuss the information sheet with the patient.

3. Confirm the inclusion and exclusion criteria.

The patient will be given time to consider the information and to ask questions. The clinician will then:

4. Obtain informed consent if the patient is willing to take part.

5. Record clinical features and whether TIA is probable or possible.

For patients diagnosed with probable TIA or minor stroke, the clinician will also undertake the following:

6. Consider the relative indications and contraindications to therapy.

7. Take the patient’s blood pressure.

8. Record any current medication.

9. Randomise the patient to the intervention or control arm.

10. Prescribe and dispense the allocated medication.

11. Ask the patient to bring their medication to the specialist clinic.

Finally, all patients will be referred to the TIA clinic with urgency determined by local guidelines. The GP will also arrange any additional appropriate follow-up consistent with usual practice.

#### Initial presentation in out-of-hours general practice

Out of the hours of 8am to 6.30pm Monday to Friday, GP care is usually provided by GPs who are not from the patient’s weekday general practice. A limited number of out-of-hours general practitioners will be trained to recruit patients into the trial. When a patient presents to a trained out-of-hours GP, the GP will take a history, examine the patient and come to an initial diagnosis of possible or probable TIA or minor stroke as per usual practice, and then:

1. Inform the patient of the suspected diagnosis as per usual practice.

2. Discuss the information sheet with the patient.

3. Confirm the inclusion and exclusion criteria.

The patient will be given time to consider the information and to ask questions. The GP will then:

4. Obtain informed consent if the patient is willing to take part.

5. Record clinical features and whether TIA is probable or possible.

For patients diagnosed with probable TIA or minor stroke, the GP will also undertake the following:

6. Consider the relative indications and contraindications to therapy.

7. Take the patient’s blood pressure.

8. Record any current medication.

9. Randomise the patient to the intervention or control arm.

10. Prescribe and dispense the allocated medication.

11. Ask the patient to bring their medication to the specialist clinic.

Finally, all patients will be referred to the TIA clinic with urgency determined by local guidelines and the daytime general practice informed by the time it reopens. The GP will also arrange any additional appropriate follow-up consistent with usual practice.

#### Initial presentation in the emergency department

Participating emergency departments will have a team of specialist nurses who support recruitment into the trial and recruitment will only occur when a specialist nurse is available. When a patient presents, the emergency department doctor will take a history, examine the patient and come to an initial diagnosis of possible or probable TIA or minor stroke as per usual practice and then:

1. Inform the patient of the suspected diagnosis as per usual practice and contact the specialist nurse.

The specialist nurse will then:

2. Discuss the information sheet with the patient.

3. Confirm the inclusion and exclusion criteria.

The patient will be given time to consider the information and to ask questions. The specialist nurse, supported by the emergency department doctor, will then:

4. Obtain informed consent if the patient is willing to take part.

5. Record clinical features and whether TIA is probable or possible.

For patients diagnosed with probable TIA or minor stroke, the specialist nurse, supported by the emergency department doctor, will also undertake the following:

6. Consider the relative indications and contraindications to therapy.

7. Take the patient’s blood pressure.

8. Record any current medication.

9. Randomise the patient to the intervention or control arm.

10. Prescribe and dispense the allocated medication.

11. Ask the patient to bring their medication to the specialist clinic.

Finally, all patients will be referred to the TIA clinic with urgency determined by local guidelines. The emergency department doctor will also arrange any additional appropriate follow-up consistent with usual practice.

#### Specialist clinic

All patients will have been referred to the TIA clinic as per local protocol consistent with NICE guidelines and therefore will usually be reviewed within 7 days of symptom onset (or within 24 hours for higher risk TIAs). Specialists will manage patients according to their usual practice, including changing any medication started by the primary care physician. In addition, the specialist doctor, supported by nursing staff, willrecord clinical features, ABCD2 score, blood pressure, final diagnosis, current medication, persistence with primary care initiated medication and changes to medication.

Additionally, the research nurse will check the patient’s hospital record for a final diagnosis when this is available if it is not confirmed during the initial TIA clinic attendance; the research nurse will also obtain an up-to-date medication list from the patient’s GP record if this has not been provided at initial presentation.

### Outcome measures

Initial assessments of outcome measures for a future RCT will be made to assess their feasibility. Additional pilot RCT outcome measures will also be recorded specifically to examine recruitment feasibility. The diagnostic accuracy study will compare the primary care physician’s and the specialist’sdiagnoses and risk stratification scores. The same outcomes will be measured for both the pilot RCT and diagnostic studies.

#### Outcome measures for the future randomised control trial

##### Primary outcome measure

The number ofstrokes within 90 days of randomisation.

### Secondary outcome measures

Clinical outcomes within 90 days of randomisation:

• All vascular events

• All major bleeding

• Death

• Ischaemic stroke

• Haemorrhagic stroke

• Fatal stroke

• Disabling stroke

• Non-disabling stroke

• TIA

We will also explore the benefit of analysing clinical outcomes at different lengths of time, including 7 days from randomisation, 7 and 90 days from first symptoms, and between randomisation and the first specialist appointment.

The definition of a TIA is ‘a transient episode of neurological dysfunction caused by focal brain, spinal cord, or retinal ischemia, without acute infarction’, with the absence of infarction supported by appropriate imaging when available [21].

#### Adverse events

Unplanned hospitalisation and falls between entry into the trial and the first specialist appointment will be reported as possible adverse events.GP contacts will be recorded. If Read codes suggest treatment has led to additional GP contacts, the research team will retrospectively analyse the GP record for further details.

#### Quality of life

Quality of life will be measured using the EQ-5D questionnaire at 90 days.

#### Cost data

A cost-effectiveness analysis will form a component of the future RCT. During the pilot phase, 90-day cost data will be gathered to develop this analysis, including:

• Drug usage

• Investigations

• Hospital admissions

• Outpatient admissions

• GP contacts

#### Process of care measures

The process of care measures are the patient’s blood pressure at 90 days and serum cholesterol at 90 days. Persistence with treatment at first specialist appointment and persistence with treatment at 90 days will be recorded.

### Additional outcome measures for the pilot randomised control trial

#### Recruitment rate

• Number assessed for eligibility by site (emergency department, out-of-hours general practice or general practice).

• Number recruited into the pilot RCT by site.

• Number who received the allocated intervention by site (including the nature of receipt of intervention group medications in the control group).

• Number followed up at 90 days by site.

• Basic demographic data and data on the presentation of the patient to the site will be collected on patients excluded or missed from the study by each site to help determine the feasibility of the trial.

• TIA clinics in participating hospitals will be audited to cross-check the data collected from each site and diagnoses of patients from participating practices will be obtained.

#### Sample size calculation

The proportion of patients experiencing a stroke within 90 days by allocation will be used to assist the sample size calculation for the future RCT. Side effects, tolerability and quality of life will be assessed from the reasons given by patients for omitting or stopping medication and their responses to the standard side-effect questionnaire at 90 days.

#### Outcome measures for the diagnostic accuracy study

• Clinic’s diagnosis of TIA or minor stroke compared with the primary care physician’s diagnosis of possible or probable TIA and with the primary care physician’s ABCD2 score.

• Clinic-defined ABCD2 score compared with primary care physician’s ABCD2 score.

• Features recognised by primary care physicians that predict TIA.

• Clinical features recognised by primary care physicians but not by specialists and vice versa.

• How well the clinical features and ABCD2 score predict for anterior versus posterior circulation events.

### Follow-up

There will be a 90-day follow-up clinic at the hospital. This will be supplemented by a review of GP and hospital records, flagging with the NHS Central Register and telephone contact or home visits for patients who do not or cannot attend the 90-day follow-up clinic. These follow-up mechanisms will:

• Confirm the final diagnosis.

• Record the patient’s blood pressure and serum cholesterol at 90 days.

• Identify primary and secondary outcomes.

• Monitor for serious adverse events and deaths.

• Record any changes in treatment and persistence with medication at 90 days.

• Collate cost data.

• Assess the patient’s quality of life at 90 days.

• Record side effects.

Prior to contacting participants for a follow-up, their health status will be established via their GPs to establish that they are not deceased and to ascertain whether their mental capacity has been lost. Participants identified as having lost capacity will be contacted and an appropriate consultee appointed. Detailed information about the trial will be provided to the consultee, and if in agreement an appointment will be made for the participant’s follow-up visit, at which the consultee must be present and must sign a declaration form to confirm their accordance.

Blood pressure and serum cholesterol readings taken at the 90-day follow-up clinic will be sent to the patients’ GP.

Where strokes are identified, anonymised copies of the relevant clinical information (from GP and hospital records, post-mortem reports, scan reports and so on) will be obtained and sent to an independent end-point committee, who will be blinded to treatment allocation. Independently of each other, the end-point committee will determine whether or not a stroke occurred, and if so, what sort of a stroke it was. Characterisation will include: ischaemic or haemorrhagic and disabling, non-disabling or TIA. Secondary outcomes will also include all vascular events, all major bleeding and deaths. Relevant clinical records will be scrutinised by an independent expert blinded to treatment allocation, who will determine whether or not a vascular event or major bleeding occurred, and what it was using standard case definitions. All hospital admissions will be scrutinised for possible adverse events and the clinical records sent to an independent expert for review.

We will follow up all patients, including those in the RCT and the diagnostic study.

### Analysis

For the statistical analysis,baseline, TIA clinic and follow-up variables will be summarised by treatment group and overall. Categorical variables will be summarised by the number and percentage of subjects in each category. Continuous variables will be summarised using the mean, median, standard deviation, interquartile range and minimum and maximum values. For the main trial, logistic regression analysis of the primary and secondary outcomes will determine the efficacy of rapid drug treatment initiation after adjusting for the variables for catchment area, clinic type and ABCD2 score. Analysis will be according to the intention-to-treat principle and the treatment effect will be expressed as an odds ratio and its corresponding 95% confidence interval. The influence of missing data on the robustness of the findings will be explored in sensitivity analyses (replacement by last value, mean of series and multiple imputation). All statistical analyses will be pre-specified in a comprehensive statistical analysis plan, which will be agreed by the trial steering committee in advance of the final database lock.

Clinical outcomes will also be analysed according to the following sub-groups:

• Specialist diagnosis of non-TIA, TIA or stroke.

• Primary care physician’s diagnosis of possible or probable TIA.

• Specialist’s ABCD2 score.

• Primary care physician’s ABCD2 score (both as recorded by the minimisation hotline and the case report forms).

• Baseline therapy.

• Recruitment site by location (Birmingham, Cambridge or Oxford) and clinic type (out-of-hours general practice, daytime general practice or emergency department).

• First TIA or stroke versus recurrent TIA or stroke.

We will analyse the TIA versus minor stroke outcome rates in both RCT groups to exclude diagnostic bias.

### Ethical approval

The RAPID-TIA protocol was granted ethical approval by the Cambridgeshire 3 Research Ethics Committee (REC reference 11 EE 0040) and is compliant with the Helsinki Declaration.

## Discussion

The main aims of the RAPID-TIA pilot trial are to determine the feasibility of a randomised controlled trial, to analyse cost effectiveness and to ask: Should general practitioners and emergency doctors initiate secondary preventative measures in addition to aspirin in people they see with suspected TIA or minor stroke at the time of referral to a specialist?

While there are potential benefits of primary care initiation of treatment, potential adverse effects need to be considered. These include the risk of extension of haemorrhagic stroke from dual anti-platelet treatment, uncertainty about the effect of early statin treatment and unnecessary treatment due to overdiagnosis.

Anti-platelet agents decrease the risk of stroke and other cardiovascular disease but increase the risk of bleeding; both of these effects are increased by dual anti-platelet agents [22,23]. Although there is controversy over whether people experiencing a haemorrhagic stroke may have their stroke worsened by anti-platelet agents [24-27], it is reasonable to be alert to the possibility that dual anti-platelets may have a poorer prognosis if they are inadvertently given to a patient experiencing a haemorrhagic stroke. However, because ischaemic stroke is substantially more common than haemorrhagic stroke, it is currently standard practice to treat patients with TIA or minor stroke with a single anti-platelet agent (aspirin) before specialist review or brain imaging, on the basis that any delay in treatment will increase the risk of recurrent stroke [6]. Patients whose neurological symptoms have not substantially resolved are at higher risk of having had a haemorrhagic event, and therefore will be excluded from this trial. Haemorrhagic strokes occurring during the pilot or main trial will be recorded as significant adverse events.

There is currently no evidence that rapid administration of statin treatment is beneficial. The FASTER study investigated treatment with simvastatin 40 mg once daily versus a placebo in 392 patients within 24 hours of a stroke or TIA and failed to demonstrate whether this was either harmful or beneficial (the absolute risk increase of stroke within 90 days was 3.3%; the 95% confidence interval was −2.3% to 8.9%; *P*=0.25) [12]. However, there is evidence that statin treatment reduces the incidence of recurrent stroke in the long term in people who have experienced a stroke or TIA [28-30] and statin treatment is recommended for all patients who experience ischaemic stroke/TIA [6].

Approximately 50% of people referred to TIA clinics have not experienced a TIA or minor stroke [31]. Common alternate diagnoses include migraine, faints, vertigo and epilepsy [32]. It is therefore likely that this trial will increase the number of people who are given blood pressure treatment, statin therapy and anti-platelet agents for the brief period (usually less than 7 days) before they are reviewed by the specialist clinic. However, all these agents are commonly used in primary care to prevent cardiovascular disease, and it is unlikely that 1 to 7 days of unnecessary treatment will cause substantial harm – indeed, since clinicians use cardiovascular risk factors to assess the likelihood of TIA or minor stroke, many patients with alternative diagnoses may benefit from these drugs and the focus on their cardiovascular risk that a suspected diagnosis brings. Significant adverse events that occur prior to specialist clinic attendance will, however, be recorded.

Given that PCPs currently refer a high proportion of patients without TIA to TIA clinics (threatening to increase the sample size requirement), this pilot study will estimate the proportion of patients with TIA who are likely to be referred in the main trial, and gather evidence to support improved diagnostic accuracy in primary care. Currently diagnostic or referral support tools have not been trialled in primary care and as such there is no current evidence base to inform an intervention to improve the diagnostic accuracy of TIA referrals from PCPs. Within the pilot study, referring PCPs will have a choice as to whether to diagnose patients as probable TIA or minor stroke (and enter them into the treatment trial) or as possible TIA or minor stroke (and not enter them into the treatment trial). It is likely that this choice will mean that more than 50% of patients entered into the treatment trial will have experienced a TIA or minor stroke, and this pilot study will gather data on whether diagnostic accuracy is higher in the “probable TIA” group.

This pilot study will therefore both test the feasibility of the main study and provide novel data on diagnostic accuracy in primary care and the primary care recorded clinical features that are associated with a positive specialist diagnosis. The main study will inform national stroke prevention policy in patients with suspected TIA and contribute significantly to the evidence base for managing TIA in primary care.

The study so far has experienced some difficulties. Recruitment occurred in real time and this can prove difficult depending on the demands the PCP is experiencing at the time of recruitment, such as a late-running clinic. This led to patients meeting the inclusion criteria being missed. We are also experiencing low recruitment rates. To try to understand why this is occurring we are carrying out audits on the participating GP practices, as well as the participating TIA clinics, to enable us to quantify the reasons why patients were not recruited into the study.

## Trial status

The study started recruiting participants in November 2011, and recruitment closed 31 December 2012.

## Abbreviations

BP: Blood pressure; GP: General practitioner; NICE: National Institute for Health and Care Excellence; PCP: Primary care physician; RCT: Randomised controlled trial; TIA: Transient ischaemic attack.

## Competing interests

The authors declare that they have no competing interests.

## Authors’ contributions

JM, RM, KF, DL, MG, DS and CW developed the original idea. JM, DE, KF, RD, RM, DL, MG, DS, GM, SC, FW and VH developed and wrote the protocol. All authors read and approved the final manuscript.
